# Complete Genome Sequence of *Chryseobacterium* sp. Strain NBC 122, a Plant Growth-Promoting Bacterium Isolated from Freshwater

**DOI:** 10.1128/MRA.01028-19

**Published:** 2020-01-23

**Authors:** Hyangmi Kim, Sang Mi Yu

**Affiliations:** a Bacteria Research Team, NNIBR, Sangju, Republic of Korea; b Environmental Microbiology Research Team, NNIBR, Sangju, Republic of Korea; DOE Joint Genome Institute

## Abstract

*Chryseobacterium* sp. strain NBC 122, isolated from freshwater of the Nakdong River in the Republic of Korea, was previously characterized as a plant growth-promoting bacterium. Here, we report the genome sequence of *Chryseobacterium* sp. NBC 122.

## ANNOUNCEMENT

According to the List of Prokaryotic Names with Standing in Nomenclature (LPSN) (www.bacterio.net/index.html), the *Flavobacteriaceae* family comprises 112 genera, including *Chryseobacterium*, which was proposed by Vandamme et al. in 1994 (1). *Chryseobacterium* species are known for their potential to promote plant growth (2, 3). For example, *Chryseobacterium* sp. strain NBC 122, isolated from freshwater samples from the Nakdong River in the Republic of Korea, has demonstrated the ability to increase the plant biomass, germination rate, and plant length of Chinese cabbage grown under saline stress and normal conditions. A patent has been granted for this bacterium for its use in enhancing the yields of Chinese cabbage (S. M. Yu, H. Kim, J. H. Eom, S. C. Jeong, W. Han, Y. T. Oh, C.-H. Kang, M. J. Kim, Y. T. Jeong, B. S. Hwang, and C. H. Kim, Republic of Korea patent registration number 1019965290000). The specific genetic determinants for the ability of *Chryseobacterium* sp. NBC 122 to increase plant biomass and the germination rate have yet to be pinpointed. Analysis of the *Chryseobacterium* sp. NBC 122 genome may help to identify the potential metabolic pathways involved in that process.

*Chryseobacterium* sp. NBC 122 was isolated from water of a branch of the Nakdong River in the Republic of Korea (36°16′08.8″N, 128°09′35.2″E). The sample was inoculated on Reasoner's 2A (R2A) agar (Difco) using the serial dilution method and was incubated at 25°C. After incubation, a single colony, designated NBC 122, was selected and routinely maintained on R2A agar at 25°C for sequencing.

The genomic DNA of *Chryseobacterium* sp. NBC 122 was extracted using a DNeasy blood and tissue kit (Qiagen, USA). Then, the genome was sequenced using both the PacBio RS II platform (Macrogen, Republic of Korea) with a SMRTbell template prep kit (Pacific Biosciences, USA) and the HiSeq X Ten with a TruSeq nano library kit (Illumina, USA) according to the manufacturer’s instructions at Macrogen (Seoul, Republic of Korea). The library insert sizes were 20 kb for PacBio RS II sequencing and 350 bp for Illumina sequencing. Overall, 85,301 long reads were obtained with PacBio sequencing, and 12,037,412 paired-end reads were obtained with Illumina sequencing. These raw sequence data were assembled with PacBio SMRT Analysis using the hierarchical genome assembly process (HGAP) version 3.0 protocol (4). To improve the genome sequence, polishing was conducted using Pilon version 1.21 with HiSeq reads. Genome annotation was performed using Prokka version 1.12b (5). All software was run with default parameters unless otherwise specified. The genome sequence of *Chryseobacterium* sp. NBC 122 consisted of a single contig of 3,139,453 bp (*N*_50_, 3,139,453 bp; coverage, 237×) with a GC content of 37.5%. It contained 3,090 coding sequences (CDS), 12 rRNA genes, and 48 tRNA genes.

### Data availability.

The complete genome sequence of *Chryseobacterium* sp. NBC 122 was submitted to the NCBI GenBank database under the accession number CP037954. The raw data were deposited under BioProject accession number PRJNA526595 and SRA accession numbers SRR10566898 and SRR10566899.
